# Advancing One Biosecurity to Address the Pandemic Risks of Biological Invasions

**DOI:** 10.1093/biosci/biab019

**Published:** 2021-03-10

**Authors:** Philip E Hulme

**Affiliations:** Lincoln University, Canterbury, New Zealand

**Keywords:** globalization, International Health Regulations, One Health, phytosanitary, Public Health Emergency of International Concern, SARS-CoV-2

The world is witnessing a global rise in numbers of emerging invasive alien species, but identifying which species pose a pandemic threat remains poorly understood. The disjointed international regulatory environment presents a significant challenge to biosecurity interventions at a global scale. A novel way forward is through One Biosecurity, an interdisciplinary approach to biosecurity policy and research that enhances the interconnections between human, animal, plant, and environmental health to prevent and mitigate the impacts of invasive alien species. One Biosecurity underpins three initiatives essential to deal with the pandemic risks from biological invasions: new risk assessment tools that look beyond national borders toward biosecurity risks of international concern, a stronger regulatory instrument to address biosecurity threats at a worldwide scale, and the establishment of a multilateral biosecurity convention responsible for biosecurity governance. Together, these initiatives will drive a new science and policy agenda to deliver evidence-based governance of global biosecurity.

The global response to prevent and contain the spread of the SARS-CoV-2 coronavirus undoubtedly represents the most dramatic biosecurity response ever undertaken at an international scale. This response contrasts markedly with the more muted international and national biosecurity strategies to manage emerging pests, pathogens, and weeds that negatively affect livestock, crops, or native species. This relative underappreciation of the risks arising from biological invasions doubtless reflects the fact that the global economic costs of recent emerging infectious diseases of humans have been huge: $40 billion for SARS-CoV in 2002–2003, $55 billion for H1N1/09 (swine flu) in 2009, and $53 billion for the Ebola outbreak in 2014 (Mackenzie 2020). Outside of human health, there are few individual invasive alien pest, pathogen or weed species that might account for such high economic costs. For example, the global annual cost of foot-and-mouth disease in livestock has been estimated to be of the order of $20 billion (Knight-Jones and Rushton 2013), whereas, for one of the most widespread insect pests of crops, the diamondback moth (Plutella xylostella), the annual costs are thought to be of the order of $4.6 billion (Bradshaw et al. 2016). But comparisons of individual taxa are misleading because the number of invasive alien pests, pathogens, and weeds worldwide is several orders of magnitude larger than the number of communicable diseases threatening human health (Seebens et al. 2017, Webber 2019). Therefore, although scenario modeling suggests that during the twenty-first century global pandemics could cost in excess of $60 billion per year (Gostin 2019), the annual cost attributed solely to invasive alien insects on goods and services worldwide is estimated to be more than $70 billion (Bradshaw et al. 2016).Despite their potentially greater economic costs, the more diffuse impacts of invasive alien pests, pathogens, and weeds, as well as their less discernible threat to human life, have resulted in a suboptimal approach to managing biosecurity risk at an international scale. The international regulatory environment addressing invasive alien species has long been recognized as ineffective as a result of strong sectorial silos that result in fractured and disjointed decision-making (Riley 2005, Shine 2007, De Poorter 2009, Outhwaite 2013, Liebhold et al. 2017). Unfortunately, the world is witnessing a global rise in the number of emerging alien species (those species never encountered as aliens before) and, in the absence of effective international regulations, these species pose a significant challenge to biosecurity interventions worldwide (Seebens et al. 2018). Inward-looking policies that solely address human, animal, plant, or environmental health are no longer fit for purpose because of the significant cross-sector impacts of invasive alien species (figure 1). Concepts such as One Medicine, One Health, EcoHealth, and Planetary Health have been proposed to bridge these sectorial divides, but they also suffer from several limitations (box 1). One Biosecurity is an alternative that provides an integrated perspective to address the many biosecurity risks that transcend the traditional boundaries of health, agriculture, and the environment including zoonotic parasites, vectors of pathogens, pests of agriculture or forestry, and threats to biodiversity (Hulme 2020).

**Figure 1.**
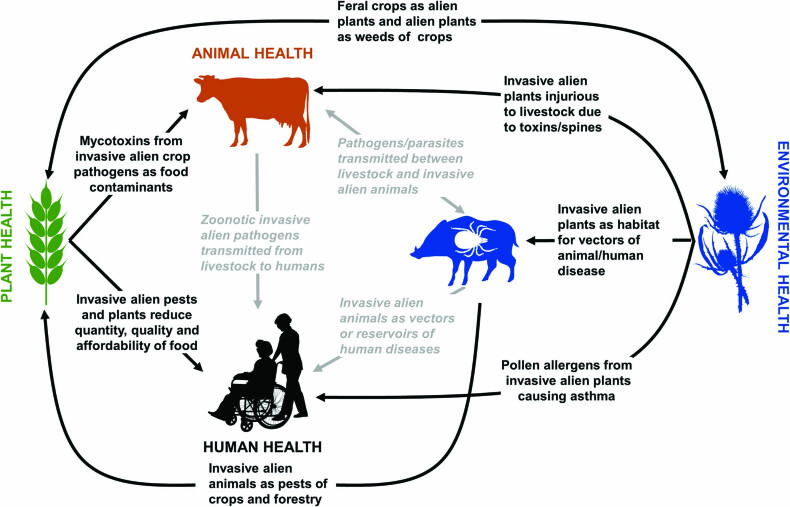
Interrelationships between human (couple with wheelchair), animal (cow), plant (wheat), and environmental (boar, tick, and thistle) health in relation to the impacts of invasive alien parasites, pathogens, insect pests, vectors, weeds, and feral vertebrates. A single example of each interaction is also provided for illustrative purposes. The figure emphasizes that a sectorial focus through the International Plant Protection Convention (plant health), the World Organisation for Animal Health (animal health), the Convention on Biological Diversity (environmental health), or the World Health Organization (human health) cannot capture the multiple direct and indirect effects of invasive alien species on human quality of life and well-being. The three links between human, animal and environmental health at the center of the figure capture the main areas encompassed by the One Health concept and highlight the much broader scope of One Biosecurity, which extends more comprehensively into plant and environmental health.

**Box 1. Progressing from One Health to One Biosecurity.**

Emerging from the idea of One Medicine, the One Health concept was formalized in 2007 with the aim of bringing veterinary and human health closer together because the divide between veterinarians and doctors was seen as an obstacle to addressing the many new or reemerging human diseases that come from animals (Monath et al. 2010). Although several definitions of One Health exist, the One Health commission (onehealthcommission.org) defines it as “a collaborative, multisectoral, and transdisciplinary approach—working at local, regional, national, and global levels—to achieve optimal health and well-being outcomes recognizing the interconnections between people, animals, plants and their shared environment.” One Health has gained considerable momentum and in 2010, the WHO, the OIE, and the Food and Agriculture Organization of the United Nations (FAO) agreed to a mandate of sharing responsibilities and coordinating global activities to address health risks at the animal–human–ecosystems interface (Khan et al. 2018). However, despite this momentum, the incorporation of environmental perspectives in One Health remains limited. For example, a systematic review of the major discipline areas covered in publications addressing “One Health” catalogued in the Web of Science between 2007 and 2020 highlights that only 7% encompass environmental sciences (see below).

Furthermore, a detailed assessment of One Health networks across the world revealed that one third don't address environmental science at all (Khan et al. 2018). It has therefore been argued that the limited engagement with environmental science severly limits the application of One Health to address the global challenges facing human well-being (Destoumieux-Garzon et al. 2018, Essack 2018). As a result, alternative concepts such as EcoHealth and Planetary Health have emerged that have a stronger emphasis on the environment but these have served only to add confusion as to the best way to address the interface between human, animal, plant and environmental health (Lerner and Berg 2017). Although wildlife reservoirs, antimicrobial resistance and zoonoses are addressed by One Health, other key isues likely to shape how the world addresses the emergence of new threats to human and animal health are poorly covered. Despite the significant role invasive alien species play in determining human and animal health outcomes (see figure 1), less than 0.5% (12 out of 3952 publications) of the literature addressing One Health examines biological invasions. Important issues that have been given scant attention in One Health (and for that matter in EcoHealth and Planetary Health) include the role of international trade and human travel, the utility of international protocols for sanitary inspections of imports and exports, the effectiveness of biosecurity interventions at international borders as well as at the farm gate, and the functionality of risk assessment tools and forecasts to predict future threats. One Biosecurity embraces these areas much more explictly and broadens the concept of One Health to include strategies and policies for mitigating risks, provides clearer emphasis on the triggers of the global spread of disease outside of endemic areas, and more explictly integrates human, animal, plant and environmental health. After more than a decade since One Health was first conceptualised, the SARS-CoV-2 pandemic has highlighted limitation to this approach and the need to embed it within a much wider framework (de Garine-Wichatitsky et al. 2020, IPBES 2020, Ruckert et al. 2020). One Biosecuirty provides such a framework and in contrast to previous concepts, sets out a possible implementation plan.

**Figure:**
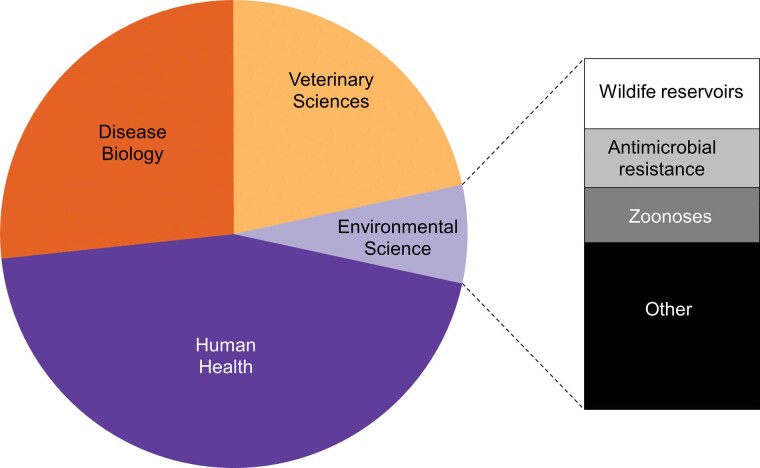
Disciplines covered in 3952 publications addressing the topic “One Health” published between 2007 and 2020 as catalogued in Web of Science.

One Biosecurity argues for greater harmonization of approaches to biosecurity threats affecting, human, animal, plant, and environmental health. At the core of the international public health response to SARS-CoV-2 is the need to address the threat of a pandemic. However, despite many invasive alien species having become established on multiple continents to the extent of effectively becoming pandemic threats (figure 2), biosecurity interventions to prevent biological invasions rarely target the risk of global proliferation. It is time for a shift in biosecurity strategies from the current focus of protecting individual countries from invasive alien species to a future emphasis on preventing the pandemic proliferation of emerging invaders across the globe. Therefore, rather than the current paradigm of preventing entry to invasive alien species through border inspections and quarantine, a more effective approach may be to prevent the deliberate or accidental export of emerging threats with potential for pandemic invasions.

**Figure 2.**
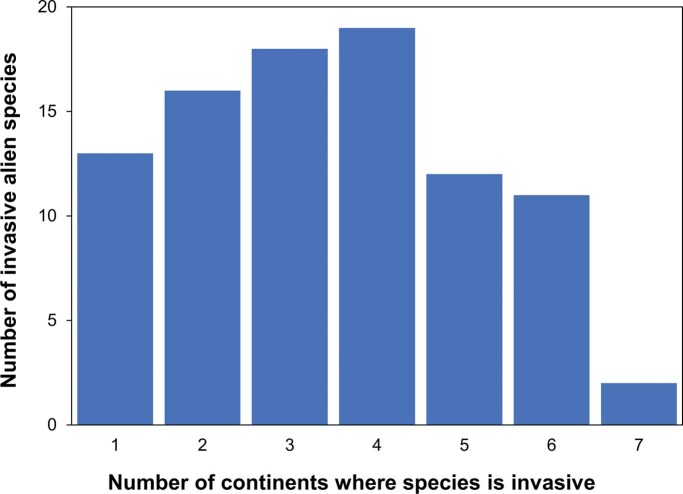
Frequency of with which species listed as among 100 of the World's Worst Invasive Alien Species (www.iucngisd.org/gisd/100_worst.php) are recorded as invasive in the Invasive Species Compendium (www.cabi.org/ISC). Although only meant to be representative, the taxa included among the 100 of the World's Worst Invasive Alien Species have been widely used to examine vulnerability of biodiversity hotspots to invasion (Bellard et al. 2014), the impact of climate change on biological invasions (Bellard et al. 2013), and regional management of invasive alien species (Faulkner et al. 2020). Only 91 of the 100 species were recorded as invasive in the Invasive Species Compendium, but over two-thirds have invaded three or more continents.

To address the pandemic risks posed by emerging invasive alien species requires fresh thinking to identify more targeted solutions to global biosecurity threats. Using the experiences of international public health in addressing recent infectious disease pandemics, three interrelated initiatives would appear essential to deal with the pandemic risks from biological invasions: an improved approach to risk assessment that looks beyond national borders toward global risk, a stronger regulatory instrument to address biosecurity threats at a worldwide scale, and the establishment of an overarching organization responsible for international biosecurity governance. The following sections discuss how each of these three initiatives might improve biosecurity strategies to limit the pandemic threat from emerging invasive alien species but also point out several important barriers to success that arise from incomplete scientific knowledge, limited global biosecurity capability, and ambivalent political support for multilateral agreements. Overcoming these barriers requires a clear roadmap based on a One Biosecurity approach that bridges the human, animal, plant, and environmental sectors. In the present article, a preliminary roadmap to the implementation of One Biosecurity is presented that sets out the advantages and challenges of different options in order to stimulate an informed debate concerning the quantum leap required to manage biological invasions effectively at a global scale. Building consensus among scientists, policymakers, and other stakeholders in terms of the best way forward is an essential first step on the long journey to designing and finally delivering a practical solution to the increasing risk of biosecurity threats worldwide.

## Evolving risk assessments to address the pandemic threats of invasive alien species

At the core of the international response to emerging infectious diseases is the obligation for countries to determine whether an event occurring within their territory might constitute a threat to the wider international community (referred to as a Public Health Emergency of International Concern, PHEIC). To qualify as such a threat, an event must meet at least two of the following four criteria: The impact must be serious, it should be unusual or unexpected, there should be a significant risk of international spread, and it could pose a significant risk to international trade or travel restrictions (Rodier et al. 2007). These criteria appear equally relevant in determining whether an invasive alien species has a high pandemic risk potential and could be classed as a Biosecurity Risk of International Concern. This would identify any emerging invasive alien species that has the potential to spread worldwide. However, the application of these criteria to biosecurity threats posed by invasive alien pests, pathogens, and weeds faces at least four major challenges to initiating a global biosecurity response.

First, in most invasive alien species incursions (with the exception of many pathogens), the species is initially identified before any impacts are documented within the territory and these impacts may occur several years after the first arrival of the species (Jarić et al. 2019). In stark contrast, often a new or emerging infectious disease is identified by its impact on human or animal health before the etiologic agent is identified. Such delays in the diagnosis of the etiologic agent of a new disease can be as brief as a few months in the case of SARS-CoV and Legionnaires’ disease, to several years for HIV, and, in some cases, such as Brainerd diarrhea, the agent remains a mystery even after several decades of research (Honigsbaum 2019, Osterholm and Olshaker 2020). As a result, public health interventions to new infectious disease threats are mobilized in response to observed impacts rather than simply the appearance of a new agent. However, even for widely established invasive alien species there remain significant gaps in the quantitative knowledge of impacts on the environment, economy, and human health (Kumschick et al. 2015). For example, using the International Union for Conservation of Nature's Environmental Impact Classification of Alien Taxa impact framework (Blackburn et al. 2014), data on the environmental impacts of invasive alien species were deficient for 18% of plant species (Rockwell-Postel et al. 2020), 24% of gastropods (Kesner and Kumschick 2018), 61% of amphibians (Kumschick et al. 2017), and 70% of birds (Evans et al. 2016). Similarly, although considerable efforts have been made to collate information on the economic costs of biological invasions, contemporary data only encompass a fraction of all invasive alien species (Diagne et al. 2020). Therefore, it is hard to forewarn the world of an impending global threat when an invasive alien species impacts have yet to be quantified (PHEIC criterion 1).

**Figure 3.**
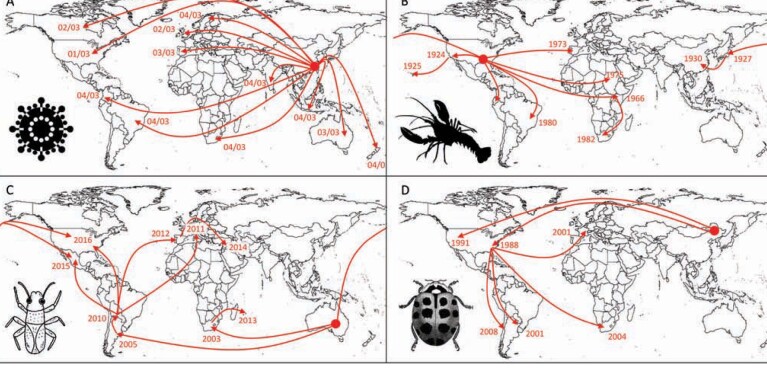
Illustration of the global patterns of pandemic spread for four invasive alien species. (a) The spread of SARS-CoV through the international movement of infected humans from China between January and April 2003 (Hempel 2018). (b) The global redistribution of the red swamp crayfish (Procambarus clarkii) as a result of escapes from aquaculture (Oficialdegui et al. 2019). (c) Worldwide dissemination of the bronze bug (Thaumastocoris peregrinus) on infested plant material to regions in which its Eucalyptus hosts have been planted commercially (Machado et al. 2020). (d) Invasion of the harlequin ladybird (Harmonia axyridis) following its global transport and deliberate releases as a biological control agent (Lombaert et al. 2010). For the sake of clarity, not all known movements of each taxon are represented on the maps. The large circle represents the approximate putative origin of the invasive alien species and the dates reflect the most likely arrival in a region.

Second, the arrival of a nonnative species known to already cause problems in other parts of the world might no longer qualify under the criterion of being unusual or unexpected (PHEIC criterion 2). Data on potential impacts of an invasive alien species are often assembled from existing literature and risk assessment tools rely heavily on evidence of invasion elsewhere (Hulme 2012). The dependence on evidence of invasive alien species impacts from other regions to signal a potential biosecurity threat of global significance may represent a situation akin to closing the stable door after the horse has bolted. The fact that a species has already caused sufficient problems in other parts of the world for its impacts to be documented would mean that the invasive alien species of interest would have already begun to spread internationally. On the other hand, any new or emerging invasive alien species threat with no record elsewhere in the world would require that the seriousness of any impacts initially be quantified through the collection of original data to confirm its threat status. This would likely lead to delays, and therefore, the subsequent information on threat status could prove less effective for early warning. This could be a significant limitation to managing future biosecurity risks given the marked rise in the number of emerging alien species (Seebens et al. 2018). Thankfully, in most cases, the global spread of invasive alien species tends to be measured in years rather than in weeks, potentially presenting a crucial window for information gathering and dissemination (figure 3). However, new risk assessment tools that are less dependent on the history of invasion elsewhere are needed.

Third, although the majority of risk assessment approaches addressing biosecurity threats evaluate the extent to which an invasive alien species might spread within a specific target region, they do not address PHEIC criterion 3 in relation to the likelihood that a species could subsequently spread internationally (Roy et al. 2018). In international public health the distinction is made between pathogens of pandemic potential and those of only epidemic or regional potential (Osterholm and Olshaker 2020). Although the precise definition of pandemic has been subject to some controversy, a standard definition is of an epidemic occurring worldwide, or over a very wide area, crossing international boundaries and usually affecting a large number of people (de Campos 2020). Despite the considerable concern invasive alien species pose to individual nations, relatively few invasive alien species have truly worldwide distributions with most restricted to one or a few regions of the world (Dyer et al. 2016, Bertelsmeier et al. 2017, Pysek et al. 2017). Nevertheless, many invasive alien species have become sufficiently widespread across multiple continents (figure 2) that they could be viewed as pandemic—for example, Siam weed (Chromolaena odorata), European starling (Sturnus vulgaris), Argentine ant (Linepithema humile), Asian kelp (Undaria pinnatifida). There have been few attempts to discern why some invasive alien species become pandemic, but recent analyses point to an interaction between life-history traits associated with environmental adaptability and strong associations with effective introduction pathways (Dyer et al. 2016, Bertelsmeier et al. 2017). Developing new risk assessment tools that address the pandemic, as well as the national risk of an incursion by an invasive alien species, would appear an essential step to identify any future Biosecurity Risk of International Concern but would also require an overhaul of existing approaches to risk analysis and management (Leung et al. 2012).

Fourth, although international trade is a major driver of the introduction and global proliferation of invasive alien species, the feedback to improved regulations to manage these biosecurity threats is weak (Perrings et al. 2010a). Invasive alien species can often be introduced to a country through trade as a contaminant of agricultural or forestry products (e.g., diseases, parasites, pests, and weeds), alternatively they may be deliberately imported as a traded commodity (e.g., pets, ornamental plants), or are introduced as stowaways (e.g., hull-fouling organisms) on or in mail, luggage, shipping containers, aircraft, and ocean-going vessels (Hulme 2015b). The effectiveness of regulatory policies addressing the risks from contaminants, deliberate imports, and stowaways differs considerably (Hulme et al. 2008). The Agreement on Sanitary and Phytosanitary (SPS) measures of the World Trade Organization (WTO) aims to protect human, animal, and plant health by reducing the threat of introducing pests and diseases of humans, livestock, and economically important plants as contaminants of traded agricultural commodities (Zahrnt 2011). In many cases, the risks to international trade posed by these contaminants of agricultural and forestry products are often mitigated by pre-export treatment (irradiation, heat, fumigation, etc.) of commodities (Follett and Neven 2006). Nevertheless, the SPS measures have led to several high profile disputes that have impeded international trade in relation to the threat posed by animal and plant diseases, as well as invasive alien pests of crops (Li et al. 2007, Higgins and Dibden 2011, Bown and Hillman 2017). Although several countries impose blacklists to prohibit the deliberate importation of harmful invasive alien species as pets or ornamental plants, these import bans usually involve few taxa and at best have a minor impact on bilateral or regional trade (Reino et al. 2017). Although many invasive alien pests and pathogens (e.g., ants, snails, moths) are transported globally as stowaways, with the exception of species associated with wood packaging and ballast water, there are few international regulations addressing how this introduction pathways might affect global trade (Hulme et al. 2008).

The foregoing highlights that, under the current regulatory environment, there are likely to be few consequences for international trade for most incursions of invasive alien species bar those that affect agriculture and forestry (PHEIC criterion 4). Furthermore, apart from the SPS agreement, much of the emphasis of the management of pathways focuses on reducing the risk of importing invasive alien species rather than the risk of re-exporting these species to other countries. As a result, invasive alien species posing pandemic risks such as the Asian house gecko (Hemidactylus frenatus) that continues to spread to countries across five continents as a stowaway in shipping containers and luggage (Hoskin 2011) or the Asian tunicate (Styela clava) that has been introduced to ports around the world as a biofoulant on the hulls of ocean-going vessels (Dupont et al. 2010) are ineffectively managed. If ports infested with biofouling species of pandemic potential were off limits to international shipping or countries with a high likelihood of exporting stowaway species were prevented from engaging in trade with countries at risk from such invasive alien species, the international dimension of managing biosecurity threats would change dramatically. Therefore, although there are certainly invasive alien species of pandemic potential that pose a risk across multiple continents, the absence of robust risk assessment tools to determine which species have pandemic potential and the lack of appropriate international regulations to limit the global spread of these species currently prevent the mounting of responses of a similar magnitude as a Public Health Emergency of International Concern.

## Building a stronger international regulatory framework for invasive alien species

The value in declaring that an emerging invasive alien is a Biosecurity Risk of International Concern is strongly dependent on a regulatory environment that facilitates a suitable response to a pandemic risk. Once again public health initiatives may provide a useful model for such an international regulatory environment. The dramatic global response to the SARS-CoV-2 pandemic has in part reflected the effectiveness of the World Health Organization's (WHO) International Health Regulations (2005), a set of legally binding obligations, that commit the 196 contracting parties to build capacity in order to prevent, protect against, control, and provide a public health response to the international spread of disease (Jenkins 2017). In particular, the International Health Regulations codified the identification and classification of a Public Health Emergency of International Concern (Gostin et al. 2020). Similarly, legal instruments bind contracting parties of the World Organisation for Animal Health (OIE) to report exceptional epidemiological events (including zoonoses) and any emerging animal diseases (Caceres et al. 2017). Driven by an understanding of One Health and the links between livestock and human infectious diseases, these two legal instruments are increasingly becoming harmonized (de La Rocque et al. 2019).

In contrast, legal instruments addressing biological invasions by invasive alien pathogens, plants, and animals are ineffective or insufficient at both the national and international levels, which has led to breaches in authority and poor enforcement capacity (Outhwaite 2017). The Convention on Biological Diversity (CBD) obliges, rather than legally binds, its 196 contracting parties to prevent the introduction of, control, or eradicate those invasive alien species that threaten ecosystems, habitats, or species (Clout and De Poorter 2005, Shine 2007). Although it is legally binding, the International Plant Protection Convention (IPPC) has an exclusive focus on plant health, and, despite the fact that the impacts on native plants are considered, the emphasis remain largely on the phytosanitary protection of ornamental, agricultural, and forestry crops (MacLeod et al. 2010). Long-standing proposals for better integration of the IPPC and OIE under either a new or an existing binding agreement to address alien animals that are not pests of plants have had little success (Shine 2007, Ormsby and Brenton-Rule 2017). Setting standards for invasive species (other than those connected to the cause and distribution of known animal diseases and zoonoses) remains outside the OIE mandate (Kahn and Pelgrim 2010).

The foregoing highlights that an instrument similar to the International Health Regulations could be a potentially powerful tool for managing the pandemic nature of a Biosecurity Risk of International Concern, not only as a result of stronger legal basis but also the reporting requirements that effectively establish a global monitoring and evaluation system. Correspondingly, there have been calls that a global framework for managing the biosecurity threats from invasive alien pests, pathogens, or weeds should be closely based on the International Health Regulations addressing infectious human diseases (Perrings et al. 2010a, Perrings et al. 2010b, Keller and Perrings 2011). These authors argued that bringing the International Health Regulations and the SPS agreement into conformity with one another would provide a means to build capability in developing countries, improve standards for the reporting of incursions and strengthen biosecurity responses, although the precise mechanism for achieving this goal has not been elaborated (Perrings et al. 2010a, Perrings et al. 2010b, Keller and Perrings 2011). However, at least three factors limit to the effectiveness of the International Health Regulations in preventing and controlling the spread of human infectious diseases and they provide important lessons for the usefulness of similar regulations for dealing with more general biosecurity pandemics.

First, despite over a decade of capacity building, only one third of countries currently meet the core capacities to implement the International Health Regulations because of insufficient resources or willingness to comply (Taylor et al. 2020). The International Health Regulations obliges member states to collaborate in mobilizing financial resources to improve their core capacity, but the regulations do not include any concrete financing mechanisms. Even in countries in which public health capability is strong, the responses to SARS-CoV-2 have been seen as inadequate (Aitken et al. 2020). Countries are generally overconfident in their ability to deal with public health emergencies and this is especially true in more developed nations (Tsai and Turbat 2020). This parlous state of affairs is likely to also be true for biosecurity capability. Indeed many countries in Africa, south and central Asia, Indochina, the Balkans, and South and Central America have limited response capacities to address biological invasions (Early et al. 2016). Although there have been repeated calls to increase the global capacity to manage biological invasions (Shimura et al. 2010, Liebhold et al. 2017, Measey et al. 2019), there have been few initiatives established to achieve this goal. It is evident that even with a legally binding international instrument, achieving such a goal would require much more investment and a longer timescale than previously envisaged. An important first step would be to undertake a systematic assessment of the operational readiness of different countries to prevent, detect, and respond to new incursions of invasive alien species, as well as the extent to which funds can be easily accessed to address biosecurity threats. Only when the extent of the global capability deficit is clearly identified would it make sense to mobilize sustained multilateral and bilateral partnerships to support low-income countries make progress with their capacity to deal with global biosecurity threats.

Second, although the obligations under the International Health Regulations were intended to facilitate global cooperation, nationalistic responses have been evident in the reactions to the declaration of a Public Health Emergency of International Concern relating to SARS-CoV-2 that have seen delays in notifying outbreaks in order to prevent potential harm to trade and tourism, as well as avoid pre-emptive restrictions against the reporting country (Gostin et al. 2020). Such nationalist reactions are not unknown in the case of outbreaks of invasive alien pests or pathogens of agricultural commodities that have led to trade restrictions and international disputes (de Miranda 2012, Cardwell and Brewin 2019). Furthermore, individual companies may fraudulently claim phytosanitary compliance by falsifying documentation (Haack et al. 2014). Unless systems are in place to deal with protectionist, fraudulent, or autocratic tendencies among nations, broadening legislation to include all invasive alien pathogens, plants, and animals could increase the risk of international disputes, encourage the imposition of further nontariff trade barriers, and disincentivise the reporting of outbreaks.

Third, although individual nations have a responsibility to detect a disease outbreak that might represent a possible Public Health Emergency of International Concern, it is down to an ad hoc technical expert group to review the available scientific evidence and assess the severity of the outbreak, its potential for international spread, and the likely impact on global trade and travel. Critics have pointed out that this process is opaque, lacks clear definitions of each of the four criteria against which a PHEIC is assessed, and often weighs political and social implications over and above technical evidence because of the perceived risk of unilateral trade and travel measures being enacted by affected countries (Mullen et al. 2020). These limitations can be addressed by designing a more robust decision-making process for determining a Biosecurity Risk of International Concern that allows for open and independent decision-making and moves away from a binary trigger to a tiered system of multiple levels of biosecurity risk to spur commensurate country responses.

Despite the logic of stronger and better coordinated legislation to address biological invasions at an international level, the world is no nearer that goal than it was a decade ago. Developing regulations along the lines of the International Health Regulations to tackle wider biosecurity threats appears to have merit, at least in terms of ensuring a formal approach to pandemic risks. However, it is evident that robust international legislation on its own is no panacea. New international legislation needs to be supported by a sizeable investment in financial and human resources to ensure global biosecurity capability is fit for purpose. Furthermore, there must be a positive economic incentive for countries to comply with any new regulatory instrument. This is where consolidated, readily accessible data on the economic costs of invasive alien species, such as that initiated by InvaCost (Diagne et al. 2020) would be especially valuable. Although it may be tempting to penalize countries for noncompliance this might only encourage them to suppress information on new incursions. Instead, those nations that have made progress with compliance despite significant economic obstacles should be rewarded through more equitable trade agreements or wider access to international markets. These issues require a more comprehensive approach to global biosecurity governance that could form the mandate of a specific organization with oversight of the implementation of international regulations and the assessment of pandemic risks.

## Establishing an organization with global oversight and governance of biosecurity

Despite facing considerable criticism (Horton 2020, Mackenzie 2020), the WHO has been the prominent international voice throughout the SARS-CoV-2 pandemic and has helped in coordinating the responses to prevent the worldwide spread of the coronavirus, mobilizing researchers, issuing regular situation reports, providing country and technical guidance, delivering travel advice and disseminating information to the wider public. Although these actions have been especially conspicuous in 2020, they reflect the standard operating procedures of global health governance that have been implemented whenever infectious disease epidemics have occurred (Ruger and Yach 2009). There is no equivalent body with an overarching governance role in biosecurity or the management of biological invasions that would develop normative instruments, lead policy dialogue, implement data collection and analysis, facilitate information exchange, and strengthen research and technical cooperation. Although some of this work may be undertaken through the CBD and IPPC there still remains limited coordination between these two conventions (Schrader et al. 2010). Furthermore, the limited progress toward internationally agreed targets to address invasive alien species (CBD 2020) indicates the need to change the status quo. But long-standing recommendations for a specific convention to address biosecurity and biological invasions (Perrings et al. 2010b, Stoett 2007, 2010) have largely gone unheeded.

In the wake of the SARS-CoV-2 pandemic, the increased understanding of the interplay between invasive alien species, public health, and food security, may provide a stronger mandate for a specific convention to address biosecurity and biological invasions. Such a convention would need to encompass an interdisciplinary One Biosecurity approach to biosecurity policy and research that builds on the interconnections between human, animal, plant, and environmental health to effectively prevent and mitigate the impacts of invasive alien species (Hulme 2020). Nevertheless, the benefits of a dedicated biosecurity convention need to be clearly articulated (box 2). This might be done effectively by using political sensitivities toward communicable human diseases as a benchmark for invasive alien species impacts. For example, in the United States the cost of a worst-case scenario Zika virus epidemic has been estimated to be $1.2 billion (Lee et al. 2017), but the current annual cost of the red imported fire ant (Solenopsis invicta) is thought to be over $7 billion (Bradshaw et al. 2016). In addition, the international dimension of the problem needs to be emphasized more strongly to point out that poor global governance means that a particular invasive alien species problem for one country today will likely become a wider problem for many countries tomorrow. There is considerable scope to leverage off the forthcoming assessment of invasive alien species undertaken by the Intergovernmental Science-Policy Platform on Biodiversity and Ecosystem Services to raise the value of a dedicated biosecurity convention (Stoett et al. 2019). Furthermore, although some authorities view biosecurity as being exclusive to the domain of biodefense and dual-use research (Hulme 2020), a multilateral biosecurity convention would have much broader scope and not touch on issues of biosafety and bioterrorism covered by the Biological Weapons Convention (Cross and Klotz 2020).

**Box 2. Mapping the long road ahead.**

With few exceptions, changes to international legislation and institutional operations tend to occur at a glacial pace, despite the best efforts of scientists. The International Convention for the Control and Management of Ships’ Ballast Water and Sediments entered into force in 2017, after three decades of science, technology and policy development, with the aim to reduce the problem of transoceanic dispersal of marine organisms in ballast water (Gollasch and David 2018). Two decades after a regional approach to tackle the problems biological invasions in Europe was recognized by policymakers, the European Union adopted its Regulation on Invasive Alien Species in 2015 that committed all Member States to manage specific species of concern (Genovesi et al. 2015). Although neither initiative is perfect (Gollasch and David 2018, Hulme 2015a, 2016), each has a strong focus on transboundary risk that illustrates a multilateral agreement addressing One Biosecurity is possible, albeit the road to implementation can take more than a decade. A major stumbling block is the nature of the current international bodies charged with the governance of human, animal, plant, and environmental health that have not kept pace with the challenges of increasing globalization and the need for cross-system thinking to address biosecurity threats. Previous proposals for binding regulations on invasive alien species have emphasized the need to manage biological invasions within a country to protect indigenous biodiversity rather than tackle transboundary risks. Such an approach has met with resistance from nation states unwilling to cede autonomy to other parties. Consequently, international organizations have primarily provided technical support, whereas nation states have retained their autonomy to manage biological invasions in their territory. Unfortunately, biosecurity threats rarely pay attention to governments or borders. For this reason, One Biosecurity requires a change in the political mindset away from such unilateralism and self-interest and toward globalism and international solidarity. There are signs that this is beginning to happen. Although sovereignty remains an important issue for nation states, recent pandemics are indicative of a normative change where the duty to report disease prevalence has often prevailed over the financial and political costs of not reporting (Davies 2012, Fidler 2003). The traditional Westphalian model of governance driven by the interest of nation states is also changing in the face of the SARS-CoV-2 pandemic that has eroded governmental authority and emphasized an increasing role of nonstate actors (e.g., NGOs, private sector entities, philanthropic foundations, and academic institutions) and global organizations in decision-making (Mackenzie 2020). Even where the response to SARS-CoV-2 has led to increased nationalist or populist rhetoric (Welfens 2020), countries are beginning to view biosecurity not merely as a domestic concern but as a foreign policy issue foundational to national security (Horton 2020). Therefore, it would make sense that negotiations over binding agreements are not left to technocrats but involve political scientists who can better understand the complexity of interstate relations, the political economy of states, different socioeconomic conditions, and geopolitical differences in response (Davies and Wenham 2020). Reconfiguring international regulations in such an environment is not going to be easy but there may be reasons for hope. One Biosecurity explicitly looks beyond national borders to the risk nation states pose to other countries. This stronger transboundary perspective differs from previous attempts to establish binding agreements on invasive alien species. As nations question the future of global governance, the opportunity for advocating for a dedicated, multilateral biosecurity convention has never been stronger.

Rather than simply acting as a clearing-house mechanism to promote technical cooperation and facilitate information exchange, the the operation of a future International Biosecurity Convention would need to be more proactive in reducing the risks from biosecurity threats. Activities would, as a minimum, include establishing and running a global surveillance and monitoring network to provide early warning of new threats, collating and curating open-access data on invasive alien species distributions, impacts on different sectors and outcomes of management programs, implementing standardize risk assessment tools, supporting and coordinating the development of game-changing management techniques (e.g., gene editing, microbial biocontrol) to combat invasive alien species of global significance, generating forecasts of future global biosecurity risks under different climate or socioeconomic scenarios, unambiguously identifying an invasive species incursion as posing a pandemic risk requiring coordinated international action, verifying compliance with norms established under the convention, negotiating with industries associated with the dissemination of potentially invasive species (e.g., pet, fur, aquaculture, hunting and fishing, garden industries) to limit such trade, instigating accredited training programs for biosecurity professionals, and mobilizing capability in times of crisis by assembling the International Biosecurity Expertise Network. This may appear a huge task, but there is already ongoing research to develop standards and protocols for global monitoring networks (Latombe et al. 2017), invasive species distribution data (Pagad et al. 2018), risk assessment tools (Roy et al. 2018), quantification of economic costs (Diagne et al. 2020), and risk forecasting (Essl et al. 2020, Seebens et al. 2020).

However, less effort has been invested in assessing and responding to the global capability deficit (FAO 2019). A key feature of the International Biosecurity Convention would need to be the ability to mobilize capability during a crisis. Across the world, there are likely to be many thousands of skilled individuals managing biological invasions for government departments or nongovernmental organizations. Such a resource represents potential standby capacity at a global scale that could be mobilized to address biosecurity threats of global significance. Experienced technical teams could be rapidly mobilized worldwide as the need arises for extended missions to provide technical support as well as on the ground coordination and assistance of response operations. For example, highly experienced helicopter pilots from New Zealand have led rodent eradications on remote islands worldwide, whereas members of the Irula tribe in India have been brought in to assist with the eradication of Burmese pythons (Python bivittatus) in Florida. Establishing the International Biosecurity Expertise Network would require the building and managing of a network of government, industry, research, and commercial providers who could be deployed in biosecurity responses worldwide. Such an audit of global expertise would be worthwhile to identify specific capability needs in different countries, taxonomic impediments to biosecurity responses, and potential complementarity of capability among neighboring countries.

A single coordinating body with a dedicated secretariat is essential to bring all these activities together but governance could take the form of a networked approach with a mix of national, regional, and international organizations, led by a high level council of countries that could make demands on individual nations that fail to comply to the norms of the convention. The International Biosecurity Convention would require an adequate budget to deliver its mandate and autonomy in how funds are disbursed. It might be expected that a more hands-on convention, rather than a technocratic body, might require at least twice the funds invested into the CBD, perhaps as much as $100 million per annum. This cost might be offset by taking on the responsibilities for biosecurity currently included in the budgets of other UN organizations. Such a proposal would require full support from the CBD, the IPPC, and the OIE and a clearer demarcation of roles in global biosecurity governance. To adequately fund the International Biosecurity Convention would require a strong case to be made not only to national governments but also the World Bank. There is an appetite for stronger biosecurity initiatives to support clean and safe trade in several countries including the United States, Australia, New Zealand, the United Kingdom, and the member states of the European Union. Building momentum among these countries to drive a dedicated convention would be an important first step. The opportunity to gain long-term financial support from major philanthropic organizations such as the Ikea Foundation, the Bill and Melinda Gates Foundation, Rotary International, and Bloomberg Philanthropies should also be explored. It is unclear to what extent biosecurity and biological invasions are even on the radar of these foundations.

## A roadmap to One Biosecurity and the management of pandemic biosecurity risks

There is now considerable evidence to support the view that the risks posed by invasive alien species to human, animal, plant, and environmental health are serious and, in the absence of coordinated action, will only become worse in the future (Pysek et al. 2020). Unfortunately, the international regulatory environment has not kept pace with these developments and is no longer fit for purpose to deal with the cross-sectorial nature of biosecurity threats. The problem of inadequate international biosecurity governance was identified by multiple authors over a decade ago (Kahn and Pelgrim 2010, Perrings et al. 2010b, Riley 2009, Shimura et al. 2010, Stoett 2010), but there has been no progress toward any resolution despite the increasing pressure arising from biosecurity threats worldwide.

**Figure 4.**
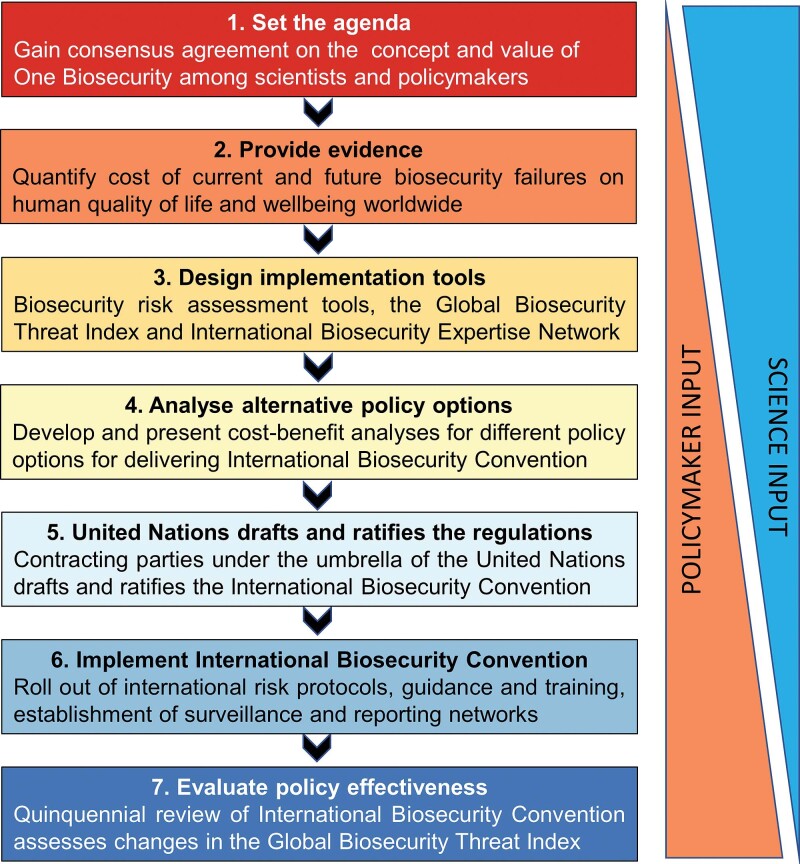
Outline of a possible implementation plan for the International Biosecurity Convention highlighting seven key steps and the relative role of scientists and policymakers at each step.

Developing a new regulatory instrument such as the International Biosecurity Convention will undoubtedly be complex but can be perceived as requiring at least seven major steps (figure 4). Initially the One Biosecurity concept must capture the imagination of scientists and policymakers alike (step 1). Adopting a One Biosecurity approach would facilitate the comparison of relative risks across the human, animal, plant, and environmental health sectors. A short-term goal should be a memorandum of understanding negotiated among the WHO, the OIE, the IPCC, and the CBD to coordinate global activities addressing One Biosecurity. Such a memorandum should explore the strong interrelationships between the impacts of invasive alien species on different sectors (figure 1). Building on the increasing public sensitivity around public health arising from the SARS-CoV-2 pandemic, communication should focus on the impact on human quality of life and well-being, be it through emerging infectious diseases and zoonoses, or other health implications such as toxins and allergens in water, food, or in the air, or via impacts on the quantity, quality, or affordability of food (Hulme 2020).

Attracting financing from governments and especially philanthropic foundations will require using a common language regarding biosecurity threats that is not sector specific but is also fundamentally human centric. This needs to be facilitated by the production of objective and reproducible data on the national, regional, and global environmental, economic, and health costs of invasive alien species presented in terms of impacts on human quality of life and well-being (step 2). This would then need to be followed up by projections of how these costs could increase in the future without the intervention of dedicated biosecurity convention to illustrate the considerable return on investment on a multilateral binding agreement. Statements regarding impacts should be put into a context that the public can relate to more personally and this means that global estimates may be less effective than national or regional cost estimates that affect the taxpayer. However, statements such as “biological invasions cost the global economy $2 trillion” while sounding impressive present the issue in abstract terms and most people find it hard to process such large sums and compare their relative value (Madison et al. 2012). Governments will first want to protect the national interest before embarking on multilateral initiative. As an example, in the United States the economic costs in terms of losses and damages of the European starling has been estimated to be $800 million, this abstract value will have more impact when it can be shown to be more than the annual public health costs of West Nile virus ($631 million) or the average hospitalization costs of influenza outbreaks ($300 million) each year (Pimentel et al. 2005).

But in addition to building the evidence base, scientists need to develop the tools necessary for implementation of a new instrument (step 3). One Biosecurity seeks the development of a suite of more holistic risk assessment tools for the comparative assessment of biosecurity threats across the human, animal, plant, and environmental health sectors. Invasion scientists need to work with veterinarians and epidemiologists to devise a standardized quantitative approach to rapidly assess actual and potential impacts of emerging invasive alien species across all sectors, rather than being sector specific (Hulme et al. 2020). In addition, these tools should capture both the epidemic and pandemic risk of emerging invasive alien species to present transparent and unambiguous assessments of invasive alien species that represent a Biosecurity Risk of International Concern. These risk assessment tools could be incorporated within a Global Biosecurity Threat Index that captures the vulnerability of nations to biological invasions and highlights how capability and infrastructural deficits could be resolved.

Because invasive alien species transcend the traditional borders of human, animal, plant, and environmental health, any development of the International Biosecurity Convention would need to work with other conventions and organizations to avoid duplication but also better coordinate activities across sectors. Therefore, the International Biosecurity Convention must be a partnership that harmonizes existing standards and protocols in such a way as to not disadvantage any one sector but also introduces a more rigorous and effective approach to global biosecurity governance and the management of pandemic threats. Existing conventions and organizations whose mandates touch on biosecurity and invasive alien species (e.g., WHO, OIE, IPPC, CBD) must recognize that the sectorial approach to managing these risks in ineffective and that a more holistic view is essential to address this problem (figure 1). However, there are multiple ways this might be achieved, and alternative models of governance would need to be explored. Some options might include a dedicated new UN organization that would oversee the International Biosecurity Convention, an existing UN organization (e.g., OIE, IPCC, CBD, WHO) that would support the new instrument, or a network of UN bodies that would cooperatively deliver the new instrument. The costs and benefits of these and other options would need to be explored in relation to delivery of specific outcomes, and the pros and cons of each option put to wider consultation (step 4). Discussion and consultation on the form of a new legally binding instrument may take several years and existing organizations or conventions may be unwilling to relinquish authority over their sectorial interests. Depending on the outcome of the analysis of different governance models, a proposal for a new legally binding instrument would need to be drafted and then ratified by contracting parties (step 5). It should be noted, however, that ratification took over a decade in the case of the International Convention for the Control and Management of Ships’ Ballast Water and Sediments (Gollasch and David 2018).

How would the International Biosecurity Convention be implemented (step 6)? Today, biosecurity threats are managed through inspection of cargo, mail, commodities, and shipping vessels; surveillance of new species incursions; and, in some cases, black or white lists relating to trade restrictions of specific species (Hulme 2011, 2014). This focus is exclusively on the risks entering a country rather than dissemination beyond national borders. These strategies would still be important in addressing pandemic risk, but they would need to be extended to support cleaner trade and human travel. For example, biosecurity authorities would have a responsibility of ensuring all containers leaving their borders were cleaned to an international standard to exclude stowaway organisms. Quaysides, warehouses, and airports dealing with commodities for export would be required to implement pest management programs using pheromone traps, bait stations, and pesticides to reduce risk of contamination of commodities (especially untreated wood products) and containers. These high risk sites for both the introduction and export of invasive alien species should form an international network that shares real-time information on interceptions and inspections of material entering and leaving individual countries and applies agreed surveillance protocols and common data standards that would be subject to third party quality control and verification. Many of these actions are extensions of current practices and should therefore be straightforward to implement. Greater effort would need to be invested in ensuring wharf structures and hard surfaces in harbors were free from high risk biofouling organisms and this would involve the development and application of environmentally friendly antifouling paints and redesign of artificial marine structures to allow effective cleaning. Perhaps most challenging would be the introduction of biosecurity screening before passenger and commodity departure rather than at the port of arrival. Under these circumstances, there would be a natural means of verification when goods or people arrived at their destination. High interception rates would point to noncompliance at the country of origin and initiate international warnings and increased scrutiny of exports and passengers from that country. Making nations, rather than individuals or companies, responsible for biosecurity breaches would strengthen the international regulatory regime.

Finally, the International Biosecurity Convention would need to be evaluated every 5 years (step 7) to ensure it remains fit for purpose. The criteria for evaluation could include progressive reduction in the vulnerabilities of countries to biosecurity threats (as measured by the Global Biosecurity Threat Index) as their national capabilities improve, a reduced rate of successive incursions of high pandemic risk pests, pathogens, and weeds from country to country (e.g., fewer cases of a Biosecurity Risk of International Concern), greater international cooperation through the International Biosecurity Expertise Network to address invasive alien species and an expanded catalogue of invasive alien species for which biosecurity risk assessments have been undertaken. It is impossible to know precisely how long each of the steps toward implementation might take but the entire process would certainly take no less than a decade and likely longer. Therefore, scientists and policymakers working with biosecurity threats must be prepared to a long-term commitment to delivering One Biosecurity.

## Conclusions

There must be a better way to manage the increased threat of invasive alien species to human health, agriculture, and the environment worldwide. A novel way forward is through the application of One Biosecurity, an interdisciplinary approach to biosecurity policy, and research that builds on the interconnections between human, animal, plant, and environmental health to prevent and mitigate the impacts of invasive alien species more effectively. At an international level, One Biosecurity leads the way to a clearer focus on the pandemic risks of invasive alien species and provides an opportunity to learn from the successes and failures of similar initiatives targeting global public health. Such a comparison highlights that the global governance of biosecurity threats requires the implementation of three interrelated initiatives: an improved approach to risk assessment that looks beyond national borders toward the pandemic risk of an invasive alien species and identifies when it might become a Biosecurity Risk of International Concern; a specific international regulatory instrument, modeled on the International Health Regulations, to enforce proactive surveillance and response to biosecurity threats worldwide; and the establishment of a dedicated, multilateral biosecurity convention responsible for international biosecurity governance. None of these initiatives will be sufficient on its own. Developing effective tools to assess the likelihood that an emerging invasive alien species poses a Biosecurity Risk of International Concern underpins the deployment of international regulations committing nations to the monitoring and reporting of these risks. Once international regulations can be agreed and ratified by a sufficient number of nations, the International Biosecurity Convention could come into force and initiate the multiple activities expected of its mandate. However, the essential groundwork for these activities such as data management systems, gap analyses of biosecurity capability, improved regional coordination of biosecurity response should be in place beforehand. These activities would give a stronger global direction to research on biological invasions, bringing different sets of expertise together across different countries in a more coordinated and applied context. Building belief among scientists, industry, policymakers, and potential funders that there is a long-term strategy to manage biological invasions will be key.

## Author Biographical

Philip Hulme (philip.hulme@lincoln.ac.nz) is a distinguished professor of plant biosecurity at Lincoln University, in Canterbury, New Zealand, and leads the biosecurity research theme in the Bio-Protection Research Centre, one of ten National Centres of Research Excellence in New Zealand.
